# Mandibular Advancement Devices With Remote Monitoring Technologies: Expanding the Dentist's Role in the Management of Obstructive Sleep Apnea

**DOI:** 10.7759/cureus.115968

**Published:** 2026-09-08

**Authors:** Joanna Guzman, Sandy Arias-Arias, Josue Santoyo, Milagros Lares, Paulina Ochoa, Gabriela Mirabal, Luisana S Rodriguez

**Affiliations:** 1 Dentistry, Universidad Central del Este, San Pedro de Macorís, DOM; 2 Orthodontics, University of Cartagena, Cartagena, COL; 3 Dentistry, Universidad Iberoamericana, Santo Domingo, DOM; 4 Dentistry, Universidad Nacional Experimental de los Llanos Centrales Rómulo Gallegos, San Juan de los Morros, VEN; 5 Dentistry, Universidad de Antioquia, Medellín, COL; 6 Orthodontics, Central University of Venezuela, Caracas, VEN; 7 Dentistry, Universidad de Carabobo, Valencia, VEN; 8 General Dentistry, Boston University School of Medicine, Boston, USA

**Keywords:** adherence, artificial intelligence, mandibular advancement device, obstructive sleep apnea, oral appliance therapy, precision dentistry, remote monitoring, smart devices

## Abstract

Obstructive sleep apnea (OSA) is a prevalent disorder with significant systemic and quality-of-life consequences. Mandibular advancement devices (MADs) are an established treatment option, but conventional follow-up often relies on intermittent visits and self-reported adherence. Sensor-integrated MADs and remote monitoring may enable objective adherence assessment and personalized longitudinal care, although their additional clinical value remains uncertain. This narrative review critically appraises current evidence on sensor-integrated MADs with remote monitoring for OSA, focusing on treatment adherence, objective monitoring, personalized therapy, and dentists' evolving role in longitudinal multidisciplinary care. We searched PubMed/MEDLINE, Embase, Scopus, and the Cochrane Library through 2025 for English-language studies on MADs, sensor-integrated oral appliances, and remote monitoring in OSA. We synthesized evidence across six domains spanning pathophysiology, clinical practice, and digital innovation. The evidence indicates that MADs improve respiratory and patient-reported outcomes, while integrating sensor-based and remote monitoring technologies offers additional opportunities for objective assessment of adherence and nightly appliance use. Remote monitoring and feedback may improve the consistency of MAD use, although increases in total nightly wearing time have not been consistently demonstrated. Emerging smart MADs can also monitor parameters such as sleep duration, sleep position, and respiratory events, while adaptive systems may enable individualized adjustment of mandibular advancement. However, current evidence does not yet establish that smart MADs provide superior long-term physiological, cardiovascular, or patient-reported outcomes compared with conventional MAD therapy. Smart MADs may nonetheless strengthen adherence assessment, expand the dentist's role in longitudinal care, and support personalized, multidisciplinary OSA management; further clinical validation and standardized long-term studies are needed to determine their incremental therapeutic value.

## Introduction and background

Obstructive sleep apnea (OSA) is a highly prevalent sleep-related breathing disorder characterized by recurrent partial or complete upper-airway collapse during sleep. Global modeling estimates that approximately 936 million adults aged 30-69 years have mild-to-severe OSA and 425 million have moderate-to-severe disease, establishing OSA as a major and frequently underdiagnosed public-health burden [1]. Continuous positive airway pressure (CPAP) remains the first-line therapy for many patients, but intolerance is common, and mandibular advancement devices (MADs) are an established alternative for those who cannot tolerate or decline CPAP. Conventional MAD care, however, has depended largely on intermittent clinical visits and self-reported wear, which limits objective assessment of adherence and treatment response.

A parallel development is the emergence of sensor-integrated, or “smart,” MADs and remote monitoring platforms, which promise objective, night-by-night data on appliance use and, increasingly, on physiological parameters and adaptive advancement. These technologies could reposition the dentist from a fabricator of a static appliance toward a coordinator of digitally connected, data-driven longitudinal therapy. Whether they improve clinically meaningful outcomes beyond conventional MADs, however, remains uncertain. This narrative review synthesizes current evidence across six connected domains: the pathophysiology and craniofacial contributions to OSA; the expanding role of the dentist; the biomechanics of MADs and their effect on the airway; smart MADs and remote monitoring; clinical effectiveness relative to conventional devices; and future directions in artificial intelligence and precision sleep dentistry.

We retrieved the literature from PubMed/MEDLINE, Embase, Scopus, and the Cochrane Library through 2025, using combinations of terms for mandibular advancement device, oral appliance, obstructive sleep apnea, sensor-integrated and smart oral appliances, remote monitoring, adherence, and artificial intelligence. We prioritized English-language systematic reviews, meta-analyses, randomized controlled trials, and observational and clinical studies, retaining earlier work where it established a mechanism or benchmark not since superseded. Consistent with a narrative design, studies were selected for their contribution to the thematic synthesis rather than through a formal systematic protocol, and no quantitative synthesis was undertaken.

## Review

This review is organized to follow OSA care from mechanism to management. It first establishes the pathophysiological and craniofacial basis of the disorder and why it falls within the dentist's field of observation, then traces the dentist's expanding role across screening, appliance selection, and follow-up. It next examines how MADs act on the airway, before turning to the sensor-integrated and remotely monitored devices that are the review's central focus, appraising their clinical effectiveness against conventional appliances and closing with emerging directions in artificial intelligence and precision sleep dentistry. Across these six domains, a consistent theme is the shift from intermittent, self-reported follow-up toward objective, longitudinal monitoring, and the corresponding evolution of the dentist from a fabricator of a static appliance into a coordinator of connected, data-driven therapy.

Pathophysiology of OSA and craniofacial contributions

OSA burden includes impaired daily functioning, increased healthcare utilization, occupational consequences, and elevated risks associated with excessive daytime sleepiness. Although obesity, age, and male sex are important risk factors, the disorder is anatomically and physiologically heterogeneous, and craniofacial structure can substantially increase susceptibility even in individuals without severe obesity. Upper-airway patency depends on the balance between collapsing intraluminal pressure and the stabilizing effects of pharyngeal anatomy and dilator-muscle activity. During wakefulness, reflex activation of the genioglossus, tensor palatini, and other pharyngeal dilators compensates for an anatomically narrow or highly collapsible airway; sleep reduces this neuromuscular compensation, particularly at sleep onset and during rapid eye movement (REM) sleep. When negative inspiratory pressure exceeds these muscles' ability to maintain lumen caliber, the retropalatal or retroglossal airway narrows or closes, producing intermittent hypoxemia, hypercapnia, and arousal in repetitive cycles of obstruction and fragmentation [2].

Craniofacial morphology influences the size of the skeletal enclosure surrounding the pharyngeal soft tissues. Mandibular retrognathia, micrognathia, maxillary constriction, and a high palatal vault can reduce posterior airway space and position the tongue closer to the posterior pharyngeal wall. Three-dimensional magnetic resonance cephalometry has shown that a smaller and shallower mandible is an independent OSA risk factor in men, while an inferior-posterior hyoid position is strongly associated with enlarged tongue volume [3]. Maxillary transverse deficiency may further narrow the nasal cavity and promote mouth breathing, increasing upper-airway resistance. These findings are clinically relevant to dentists because mandibular dimensions, transverse maxillary relationships, palatal morphology, and tongue position are routinely observable during dental and orthodontic examination.

The tongue is a particularly important dynamic determinant of obstruction. During sleep, reduced genioglossal tone permits posterior displacement of the tongue base, especially when mandibular support is deficient. Magnetic resonance imaging demonstrates greater tongue volume and increased tongue-fat deposition, particularly at the tongue base, in individuals with OSA compared with body-mass-index-matched controls [4]. Airway obstruction therefore reflects the interaction of skeletal restriction, soft-tissue volume, body position, and impaired neuromuscular responsiveness rather than a single anatomical abnormality.

Untreated OSA has consequences beyond sleepiness and snoring. Recurrent hypoxemia, sympathetic activation, oxidative stress, and intrathoracic-pressure swings contribute to hypertension, arrhythmias, coronary disease, and stroke; long-term observational data show increased fatal and nonfatal cardiovascular events in men with untreated severe OSA [5]. Population-based evidence also associates sleep-disordered breathing with glucose intolerance and insulin resistance independent of major confounders [6], and repeated arousals and hypoxemia impair attention, memory, executive performance, and overall quality of life, with multicohort data linking OSA to poorer global cognition [7]. Recognition of these anatomical and systemic relationships places dentists in a strategic position to identify craniofacial risk markers, initiate appropriate referral, and participate in multidisciplinary OSA management.

The expanding role of the dentist in OSA diagnosis and management

The scope of dental practice in OSA has broadened beyond constructing and titrating MADs to a more integrated role within multidisciplinary sleep medicine. The contemporary dental clinician is called upon not only to fabricate and adjust oral appliances but also to recognize patients who may be at risk, evaluate craniofacial and dental candidacy for oral appliance therapy (OAT), monitor the physiological and structural effects of treatment, and maintain ongoing collaboration with sleep physicians [8-10].

Screening within the dental setting is an essential entry point into the OSA care pathway. Routine appointments allow clinicians to observe or elicit signs consistent with the disorder, including pronounced snoring, witnessed breathing pauses, marked daytime fatigue, recurrent morning headaches, nocturia, obesity, and hypertension refractory to standard treatment. Tools such as the STOP-BANG questionnaire or the Epworth Sleepiness Scale (ESS) can assist in stratifying risk; however, such screening is preliminary and does not substitute for formal diagnosis, and any patient in whom OSA is suspected should be directed to a physician or sleep medicine specialist for definitive evaluation, including objective sleep testing [8,10]. Beyond screening, a detailed clinical and airway examination should address craniofacial structure, mandibular positioning and mobility, tongue and palate anatomy, tonsillar size, nasal patency, occlusal relationships, periodontal condition, dental stability, and temporomandibular joint function, because the success of MAD therapy depends on sufficient dental support and the patient's ability to tolerate mandibular protrusion. Comparative evidence further shows that custom-fabricated devices outperform thermoplastic alternatives, reinforcing the value of individualized assessment [11].

Dental involvement should not proceed without the sleep physician's oversight. Guidelines from the American Academy of Sleep Medicine and the American Academy of Dental Sleep Medicine recommend custom, adjustable oral appliances for suitable adult candidates, especially those unable to tolerate CPAP or who prefer an alternative; within this shared model, the dentist selects, fits, adjusts, and monitors the appliance, while diagnosis and confirmation of treatment success through sleep testing remain within the medical domain [9,10]. Selecting suitable candidates requires attention to disease severity, patient preference, craniofacial structure, dental and periodontal condition, mandibular range of motion, and temporomandibular joint health, and certain factors limit or preclude the approach, including inadequate dentition for stable retention, advanced periodontal breakdown, notable temporomandibular dysfunction, restricted jaw movement, and poor tolerance of advancement; patients with severe disease demand heightened caution and tight coordination with the sleep physician [9].

Once an appliance is delivered, the dentist assumes considerable responsibility for continued care: gradual titration, evaluation of comfort and adherence, tracking of dental and occlusal shifts, attention to temporomandibular symptoms, inspection of appliance condition, and management of adverse reactions. A notable shift is underway in how this follow-up is conducted, from periodic in-office visits toward continuous, remote observation. Work by Kwon et al. has shown that remote monitoring paired with patient feedback can reliably track objective MAD compliance, pointing toward a more anticipatory approach to dental sleep medicine [12]. Digital tools reinforce this shift: intraoral scanners, computer-aided design and manufacturing, electronic recordkeeping, and virtual communication support more precise fabrication and smoother coordination, while a newer generation of sensor-equipped smart MADs can track adherence and other parameters directly, and some active devices adjust advancement based on patient data [13]. Remote titration is also becoming practical, with home-collected monitoring data relayed to clinicians in real time to guide adjustments [14].

MADs: biomechanics, tongue position, and airway remodeling

MADs function as intraoral biomechanical appliances: protrusive force is transmitted through the dentition to the mandible, which repositions the soft tissues anchored to it. Because the genioglossus and suprahyoid musculature arise from the mandibular symphysis, forward displacement of the jaw draws the tongue base away from the posterior pharyngeal wall and stabilizes the retrolingual segment during sleep. Magnetic resonance imaging has clarified how this translates into airway change: among 69 patients whose apnea-hypopnea index (AHI) fell from 27.0 to 12.2 events/h, appliance insertion enlarged total upper-airway volume, with the velopharynx contributing most of the gain, alongside increased lower anterior facial height, a shorter hyoid-to-posterior-nasal-spine distance, lateral displacement of the parapharyngeal fat pads, and anterior movement of the tongue-base musculature; importantly, this enlargement was confined to treatment responders [15].

Biomechanical and dose-response effects

Physiological work confirms a graded effect. When protrusion was advanced during sleep to 0%, 50%, and 100% of maximum comfortable protrusion, pharyngeal critical closing pressure fell in a dose-dependent fashion, whereas genioglossal responsiveness to negative pressure showed no systematic change, indicating that the dominant action is mechanical rather than neuromuscular [16]. Stepwise titration in 0.5 mm increments produced parallel dose-dependent gains in AHI improvement, success, and normalization rates, all plateauing near 70% of maximal protrusion, with effective target protrusion averaging 3.5 mm in mild, 5.8 mm in moderate, and 5.9 mm in severe disease [17]. Meta-regression across randomized trials nevertheless shows that AHI reduction is not strictly proportional to the millimeters advanced, favoring individualized titration over maximal protrusion [18].

Design variables appear less decisive than protrusion itself. A systematic review with meta-analysis of randomized trials could not identify any single construction as superior for AHI reduction, leaving the choice between custom and prefabricated, titratable and fixed, or monobloc and bibloc appliances largely a matter of retention, tolerance, and adjustability [19]. Consistent with this, a randomized trial comparing devices allowing limited versus unrestricted vertical opening found equivalent respiratory and cone-beam computed tomography (CBCT) airway outcomes in mild-to-moderate OSA [20]. Assessment methods remain complementary rather than interchangeable: CBCT reliably quantifies volumetric and minimal cross-sectional change, yet awake measurements did not discriminate responders from non-responders [21], and drug-induced sleep endoscopy addresses that limitation by visualizing collapse under sedation, where an interim appliance and a jaw-thrust maneuver reduced obstruction by 54% and 57%, respectively, supporting endoscopy as a pre-treatment selection tool [22].

Biomechanical efficacy carries a dental cost, since reaction forces are dissipated through the supporting teeth. Salivation, transient occlusal awareness, and muscular or temporomandibular tenderness dominate the first weeks and are generally self-limiting. Longer-term pooled data document progressive reduction of overbite (0.87 mm) and overjet (0.86 mm), with retroclination of the maxillary incisors and proclination of the mandibular incisors, while skeletal change remains non-significant and the magnitude increases with treatment duration [23]. Understanding these mechanics obliges the dentist to titrate to the minimum effective protrusion and monitor occlusion as therapy continues, a responsibility that becomes considerably more tractable when appliances report how and how often they are worn.

Smart MADs: remote monitoring and digital health integration

Smart MADs represent a technological evolution in OSA treatment. Conventional therapy generally relies on scheduled follow-up and manual adjustment, whereas smart MADs incorporate technologies that objectively collect and evaluate information during treatment; they may function as passive systems that record treatment-related data through embedded technologies or as active systems that use acquired information to modify advancement in response to changing conditions during sleep [13]. Integrated sensing is central and may include temperature sensors, accelerometers, magnetometers, micro-recorders, and internal data storage, enabling the capture of clinically relevant parameters such as appliance use, adherence, sleep duration, body position, and respiratory activity [13,24]. Temperature-based microsensors can document the frequency and duration of use over prolonged periods, providing an objective adherence measure that does not depend exclusively on patient report [25].

Integration with digital health technologies further expands longitudinal monitoring. Wireless communication, mobile applications, and cloud-based platforms can transmit and enable remote review of information collected during nightly use, and some systems deliver automated reminders or feedback when use is absent or insufficient. Evidence suggests that remote monitoring and feedback may increase the number of days on which patients use their appliance, although improvements in total nightly wear have not been consistently demonstrated [12]. These findings indicate that adherence is multidimensional and that remote monitoring may be particularly valuable for identifying specific patterns of use rather than categorizing patients simply as adherent or non-adherent.

Recent developments are moving smart MADs beyond data collection toward devices that adapt treatment based on information obtained during sleep, although these systems remain at different stages of technological and clinical development, and evidence of additional therapeutic advantage over conventional MADs is still emerging [13]. Auto-titrating approaches illustrate this potential by modifying protrusion based on sleep position, providing greater advancement in the supine position and reduced protrusion during lateral sleep [26]. Such systems may also expand the dentist's involvement by supporting continuous, data-informed follow-up, facilitating earlier recognition of inadequate adherence or insufficient response and better communication with sleep physicians; nevertheless, current evidence is heterogeneous, and further validation is required to establish the accuracy, reliability, clinical relevance, and long-term utility of these technologies [13,24].

Clinical effectiveness: adherence, airway outcomes, and long-term success

The key clinical question is whether smart MADs provide measurable benefits beyond conventional therapy. Current smart-MAD research favors technologies that collect adherence data, while relatively few studies have tested whether these added functions improve outcomes [13]. Conventional MAD therapy provides the benchmark: in a 2024 meta-analysis, MADs reduced AHI by approximately 58%, 67%, and 66% in mild, moderate, and severe OSA, respectively, with improvements also in oxygen desaturation index (ODI), lowest oxygen saturation, and ESS scores [27]. Long-term MAD treatment was associated with sustained reductions in AHI, ODI, and ESS, although pooled Pittsburgh Sleep Quality Index scores and sleep efficiency did not improve significantly [28]. Sleep-related functional quality of life improved with OAT, although benefits were inconsistent across the instruments evaluated [29].

Adherence is central to real-world effectiveness. A systematic review of 32 randomized trials reported an overall dropout of approximately 17%, increasing over time, with custom-made duo-bloc MADs showing greater nightly use than non-custom devices, though much of the adherence literature relied on self-report and may overestimate use [30]. In a randomized crossover study using an embedded recorder, digital monitoring and feedback increased MAD use from 4.7 to 5.4 nights per week, whereas time worn on nights of use remained essentially unchanged, and self-reported use exceeded objectively recorded use by an average of 18 minutes [12]. Newer smart MADs may extend monitoring beyond adherence: in a pilot validation study, sensor-derived estimates of treatment AHI and total sleep time closely tracked polysomnographic measurements while the device also recorded usage and sleep position, supporting the feasibility of nightly objective assessment but not establishing that sensor integration itself improves respiratory or long-term outcomes [24].

Cardiovascular evidence remains cautious. A 2024 meta-analysis found no significant overall reduction in systolic or diastolic blood pressure versus control, although meta-regression suggested greater systolic effects at night and with prolonged treatment [31], and long-term evidence suggests possible blood-pressure benefit with limited certainty [28]. Direct comparative evidence for patient-reported outcomes and the incremental cost-effectiveness of smart MADs remains limited, and standardized patient-reported measures and cost-effectiveness are research priorities [13]. Overall, smart MADs currently show their clearest added value in objective monitoring and potentially in improving consistency of use; whether these capabilities translate into superior physiological, patient-reported, cardiovascular, or long-term outcomes compared with conventional MADs remains uncertain.

Future directions: artificial intelligence, precision sleep dentistry, and personalized therapy

MAD management is evolving from a one-size-fits-all model toward a precision-medicine approach based on the P4 principles of prediction, prevention, personalization, and patient participation [32], grounded in the recognition that OSA is a heterogeneous disorder whose phenotypic and endotypic diversity determines who benefits from intraoral device therapy [33]. Artificial intelligence and advanced analytics are increasingly applied to characterize the pathophysiological traits that predict response to MADs. Four main endotypes, namely, impaired upper-airway dilator muscle function, low respiratory arousal threshold, high loop gain, and anatomical compromise, influence outcomes in distinct ways, and patients with a favorable response tend to exhibit a lower REM-associated AHI, mandibular retrusion, and reduced pharyngeal muscle compensation [33]. Complementary evidence from a large prospective cohort showed that combining hypoxic burden and positional OSA yielded a model with 96.4% predictive accuracy (area under the curve 0.98) for treatment response, underscoring the value of integrating polysomnographic phenotyping into decision algorithms [34].

Parallel advances in sensor technology are reshaping remote monitoring. A systematic review of smart MADs identified a growing distinction between passive, thermosensor-based compliance trackers and active, feedback-controlled positioners that adjust protrusion in real time based on oxygen desaturation data; although most evidence remains observational and active devices are still at intermediate technological readiness, the trajectory points toward oral appliances that continuously measure oxygen saturation, respiratory rate, and sleep stage, mirroring platforms already established for CPAP [13,32]. Digital manufacturing is simultaneously transforming customization: fully digital, single-visit workflows combining intraoral scanning, digital design, and in-office three-dimensional printing enable fabrication of adjustable, interlocking devices in hours rather than weeks and at a fraction of the conventional cost, with clinical data showing significant reductions in the respiratory event index, favorable safety, minimal one-year dentoalveolar change, and high adherence and satisfaction [35,36].

Looking ahead, the convergence of endotype-based selection, artificial-intelligence-assisted predictive modeling, and connected oral appliances is expected to enable dentists and sleep physicians to move beyond empirical titration toward individualized combination therapies [32]. Significant barriers remain, however, including prospective validation of endotyping algorithms in larger cohorts, standardization of outcome metrics for smart devices, and resolution of data-privacy and security concerns in cloud-connected systems [13]. Addressing these issues will be essential to translating the promise of artificial-intelligence-driven precision sleep dentistry into reproducible practice, and ultimately points toward a redefinition of the dentist's role, from a clinician who fabricates a static appliance to one who manages a digitally connected, data-driven therapy, interpreting phenotyping data, analyzing sensor-derived adherence trends, and coordinating personalized care with sleep medicine specialists.

## Conclusions

Smart MADs extend established oral appliance therapy by adding objective monitoring, remote follow-up, and potential adaptive treatment. Their clearest current value lies in objectively documenting adherence and appliance use and in improving the consistency of nightly wear, capabilities that strengthen the dentist's ability to conduct anticipatory, longitudinal follow-up and to communicate effectively with sleep physicians within a multidisciplinary model. These strengths, together with advances in endotype-based patient selection, artificial-intelligence-assisted prediction, and single-visit digital manufacturing, outline a credible path toward personalized, precision sleep dentistry in which the dentist manages a connected, data-driven therapy rather than a static appliance.

Superiority over conventional MADs nonetheless remains unproven. Current evidence does not establish that sensor integration or remote monitoring produces better long-term respiratory, cardiovascular, or patient-reported outcomes, and much of the supporting data is observational, short-term, or derived from devices at intermediate stages of development. Standardized long-term clinical studies, validated patient-reported and cost-effectiveness measures, prospective testing of predictive algorithms, and attention to data privacy are therefore needed before these technologies can be considered a routine advance over conventional therapy. Until such evidence exists, smart MADs are best regarded as a promising extension of oral appliance therapy that expands the dentist's role in OSA management without yet replacing the established conventional device.
